# Prayers for Sickness: What do people pray for and how do they deal with unanswered prayer?

**DOI:** 10.12688/f1000research.145194.1

**Published:** 2024-03-01

**Authors:** Simon Dein, Candy Gunther Brown

**Affiliations:** 1 Queen Mary University of London, London, England, UK; 2 Indiana University Bloomington, Bloomington, Indiana, USA

**Keywords:** Prayer, miracle, illness

## Abstract

**Background:**

This paper focuses upon prayer for sickness. What do individuals suffering from illness, their families and the wider community pray for? How do they deal with unanswered prayer? Do they pray for cure, to guide medical professionals or to cope with their sickness? What rationalisations do they proffer for unanswered prayer?

**Methods:**

Based on a critical literature review and deploying secondary data from the Twenty First Century Evangelical research programme, the data suggest that prayers for guiding medical professionals and coping are more common than for cure, at least in Global North countries such as the UK and US. But why do those who believe in miracles not ask God for divine healing? Furthermore, unanswered prayer can conflict with Christian views of God as omnipotent and all loving.

**Results:**

Respondents use a number of theodical rationalisations to resolve this conflict.

**Conclusions:**

The results are discussed in relation to cognitive dissonance theory, learned helplessness, the need to conserve a relationship with the Divine, and desire to manage risk of disappointment and reduce consequent emotional pain.

What things soever you desire, when you pray, believe that you receive them, and

Therefore I tell you, whatever you ask for in prayer, believe that you have received it, and it will be yours (Mark11:24).

The use of prayer at times of sickness is commonplace ( Schmied, 2002; Ap Siôn, 2007). There is evidence that prayer is commonly employed to cope in illness related contexts and is widely used by individuals with a broad range of illnesses ( Saydah & Eberhardt, 2006): sickle cell disease ( Ohaeri *et al.*, 1995), arthritis ( Boisset & Fitzcharles, 1994; Matthews *et al.*, 2000), HIV/AIDS ( Carson, 1993; Pargament, McCarthy & Shah, 2004) and renal disease ( Gurklis & Menke, 1988). It is a common coping strategy in cancer ( Meraviglia, 2002; Taylor & Outlaw, 2002; Peteet & Balboni, 2013; Taylor *et al.*, 1999; Gall & Cornblat, 2002; Meraviglia, 2002). Prayer has been found to have both a positive ( Maltby Lewis & Day, 2008; Francis & Kaldor, 2002) and negative effect on mental health ( Koenig, 1998; Andersson, 2008; Rippentrop *et al.*, 2005).

While intercessory prayer for illness has received significant academic attention, the results are inconclusive and inconsistent. The majority of studies demonstrate no improvement compared with those not prayed for; however, the limitations of such studies are so significant that it should not be concluded from this research that the prayer is ineffective. As the authors of a Cochrane systematic review of intercessory prayer ( Roberts *et al*., 2009, 15-16) concluded, “the studies that have been done, reported and included in this review do not show an effect of intercessory prayer. However, because this review highlighted no clear effects does not mean that intercessory prayer does not work. The limitations in trial design and reporting are enough to hide a real beneficial effect and we found no data to contraindicate the use of prayer for seriously ill people.” For example, one of the studies included in this Cochrane review ( Benson *et al*., 2006) has such serious problems with both ecological validity and construct validity that it is unclear whether the study results contribute anything to understanding generalizable effects of intercessory prayer. Most people who pray expectantly for healing—and report healing as an effect of their prayers pray—are not praying at a “distance” for people they have never met; unlike most of the intercessors recruited by Benson, they do conceive of themselves as praying to a deity outside themselves who is willing and able to answer prayer and they do consider healing to be a legitimate objective of prayer ( Brown, 2012). Furthermore, the studies are plagued with methodological and ethical issues such as difficulties achieving a pure control group, what constitutes a ‘dose’ of prayer and the theological view that God should not be subject to empirical testing ( Vincent, 2016; Masters, Spielman & Goodson, 2006; Dein, 2019).

Clinical studies of proximal intercessory prayer have been better designed than studies of distant prayer in terms of ecological and construct validity, although it is more difficult to control for potential confounds such as the well-known ‘placebo effect’ ( Hróbjartsson & Gøtzsche, 2010); clinical research on proximal intercessory prayer does suggest that patients experience health benefits from prayer ( Matthews *et al.*, 2000; Brown *et al.*, 2010). Moreover, case studies ( Romez *et al.*, 2019; Romez *et al.*, 2021) report on individuals who have experienced medically remarkable recoveries associated with proximal intercessory prayer.

## Miraculous healing

The belief that God can bring about a miraculous healing is ubiquitous in the Bible and prevalent among contemporary Christians. There is a widespread belief among Christian believers that God is able to heal sickness and often does so. Prayer requests reflect this ( Pawlikowski *et al.*, 2015; Levin, 2016).

Village (2005) notes how there are diverse views concerning miraculous healing among Christians- including beliefs about the possibility of God healing miraculously, the relationship between divine healing and secular medicine and God’s ability to heal. There may even be a diversity of views among members of the same congregation. Groups differ in how they conceptualise God working through ‘natural’ channels. As Village argues, ‘Upholding the sovereignty of God in such circumstances seems to naturally turn attention to the sufferers or to those doing the praying as possible reasons for unanswered prayer’ (p.105). Differing attitudes towards miracles among Christians might reflect diverse understandings of the Divine and the relationships between God and the natural world. Fairly widespread beliefs in miraculous healing are found among Pentecostal and Charismatic groups ( Singleton, 2001).

Candy Gunther Brown (2012) notes how Pentecostals underscore the healing power of the Holy Spirit both for physical and for psychological problems. Healing is a central aspect of the Pentecostal movement and can account for its widespread appeal, and particularly its growth in the Global South – areas of Latin America, Africa, and Asia where poverty-related infirmities are ubiquitous and conventional medical treatments limited ( Brown, 2011). Pentecostals deploy prayer alongside biomedicine rather than as a substitute for it. While testimonies of divine healing are commonplace among Pentecostals, they are often reluctant to subject these claims to empirical scrutiny:

‘Although blatantly anti-medical stances have become much less common among Christians in recent years, many Christians continue to feel ambivalent about modern medicine. Christians want the benefits of medical technology, but they also worry that relying on medicine too much, or seeking medical proof that God heals, hinders faith in God. There is residual unease with biomedicine’s materialistic assumptions, interventionist approach, and apparent hubris.’ ( Brown, 2022:5).

As Brown states in an interview for *The New York Times* :

If God can heal, why does he do it so rarely? The world is full of suffering people who pray with no relief. “Even people who believe in miracles often don’t pray for them because they’re afraid of disappointment,” Candy Brown said. “I’ve had people die on my watch. It’s incredibly painful. You ask, ‘Is it my fault?’” She speculated that many Christians’ belief that miraculous healing ceased after New Testament times springs from “protection against pain, protection against feeling ill will toward God or other people. It takes hope and vulnerability to be open to healing.” ( Kashdan *et al.*, 2006)

Further evidence that belief in Divine healing is widespread derives from a Barna study in the USA ( Barna, 2016) which indicated that the majority of American adults (66%) held that people could be physically healed through supernatural means by God (strongly agree 33%; somewhat 33%). Evangelicals were the most likely to endorse Divine healing. Respondents who disagreed were comprised of those who either strongly (19%) or somewhat (15%) disagree. Millennials and those who were more highly educated were significantly more likely to be sceptical. Of significant interest Protestants were almost three times more likely to believe people could receive divine healing than Catholics. In this survey 68% of adults had personally prayed for supernatural healing for another person.

A ten-country study ( Lugo *et al*., 2006) found widespread belief in the efficacy of prayer for healing, especially among Pentecostal Christians; 77% of Brazilian Pentecostals, 87% of Kenyan Pentecostals, 74% of Indian Pentecostals, and 62% U.S. Pentecostals claimed to have “witnessed divine healings.” Some scholars estimate that in regions, such as East Asia, where pentecostalism is growing the more rapidly, as many as 80-90% of conversions result from first-hand experiences with healing prayer ( Oblau, 2011; Keener, 2011, 2021). Globally, pentecostalism grew at four times the rate of global Christianity and the total world population between 1970 and 2010, such that there are now an estimated 635 million pentecostals worldwide ( Johnson & Zurlo, 2020). It is even more noteworthy that 73% of US medical doctors report believing that miraculous healing continues to occur in the present ( Orr, 2008).

Once a common ritual among Christians, the rise of scientific medicine has marginalised prayers for healing, at least in the Global North ( Simpson, 1993). A BBC survey ( Strangwaves-Booth, 2018) found 62% of adults in Britain expressing belief in the possibility of miracles; although 59% of Christians in the UK report praying for miracles, only 29 percent maintain that their prayers are answered. However it is acknowledged, based upon biblical teaching, that God may not answer every prayer for sickness. As an example David’s son died of an illness despite his father’s prayers. 2 Samuel 12 describes how King David fasted and prayed for seven days for God to spare his dying child but he subsequently died. In verse 22, David said, “Who knows? The Lord may be gracious to me and let the child live.” In this particular example, the text explains the death as a direct result of David’s sin in begetting the child through an adulterous affair concealed by murder. Unanswered prayer is often put down to lack of faith or sin:

James 4:3 **‘**You ask and do not receive, because you ask wrongly, to spend it on your passions’

Matthew 21:22 ‘And whatever you ask in prayer, you will receive, if you have faith.”

1 Peter 3:12 For the eyes of the Lord are on the righteous, and his ears are open to their prayer. But the face of the Lord is against those who do evil.”

It is more common among pentecostals in the Global South to emphasize the Bible’s promises of healing through prayer and to read these literally as a kind of “how-to” manual for the present day ( Jenkins, 2008). Thus, they emphasize verses such as Matthew 4:23: “Jesus went throughout Galilee, teaching in their synagogues, proclaiming the good news of the kingdom, and healing every disease and sickness.” They are confident that healing is for today based on verses such as Hebrews 13:8: “Jesus Christ is the same yesterday and today and forever.” They point to instructions to the Christian such as James 5:14-16: “Is anyone among you sick? Let them call the elders of the church to pray over them and anoint them with oil in the name of the Lord. And the prayer offered in faith will make the sick person well.” For these pentecostals, references to the “prayer of faith” is not so much an explanation for why prayers might fail as an exhortation to pray all the more: “Will not God bring about justice for his chosen ones, who cry out to him day and night? Will he keep putting them off?” (Luke 18:7).

## What do believers ask God for in relation to sickness?

While much of the literature has focused upon prayer in relation to psychological and physical outcomes, there is a small literature asking about the content of prayer. Sociologist of religion, Robert Wuthnow (2008), notes that while prayer is widely practiced, it is treated mostly without taking account of its cognitive content or cultural framing. There has been little attention devoted to the content of prayer, other than the assumption that it involves petitions. Byrd’s study of distant intercessory prayer among patients suffering from cardiac problems limited intercessors accepted into the study to “born again” Christians praying “to the Judeo-Christian God.” The pentecostals studied by Brown (2011, 2012) begin with the theological premise that prayers should be “in the name of Jesus by the power of the Holy Spirit.” The intercessor may “petition God to heal, invite the Holy Spirit’s anointing, and/or command the healing and departure of any evil spirits in Jesus’ name” ( Brown *et al.*, 2010: 865). Romez (2019, 291) described a prayer intervention credited with an instantaneous healing of gastroparesis (paralyzed stomach) in this way: “The intercessor prayed that, in the name of Jesus, the boy’s stomach be healed. He commanded the healing in the authority and power of Jesus. He made a point of indicating that he had no power or authority to heal, but only with the authority of Jesus Christ, he could command the healing.” Romez (2021, 80) similarly described a prayer credited with instantaneous healing of blindness: “Oh God! You can restore … eyesight tonight, Lord. I know You can do it! And I pray You will do it tonight.” Pentecostals believe moreover that not only is the language of prayer important but that certain individuals may have specific “gifts” of healing, sometimes even “specializing” in particular conditions such as rheumatoid arthritis or deafness ( Matthews, 2000; Brown *et al.*, 2010). In all such instances, disease and other infirmities are envisioned as enemies against which the Christian battles in prayer rather than as “blessings” sent by God for the Christian’s spiritual benefit. Accordingly, although these Christians do petition God to heal as well as commanding diseases and demonic spirits to go, petitionary prayers are not phrased “if it by Thy will” but in confidence that healing *is* God’s will; “A man with leprosy came and knelt before him and said, “Lord, if you are willing, you can make me clean.” Jesus reached out his hand and touched the man. “I am willing,” he said. “Be clean!” Immediately he was cleansed of his leprosy” (Matthew 8:1-3). Pentecostals are wont to note that the Greek word ( *thelo*) translated as “willing” does not connote reluctance, but intense desire, resolution, and delight ( Gulick, 2013).

Jors *et al.*’s (2015) systematic review of personal prayer at times of sickness included a wide range of diseases: paediatric sickle cell, cardiac surgery, prostate cancer, HIV, breast cancer, chronic hepatitis C. The final study included 16 articles and indicated that there were five main reasons for and topics of prayer: (1) disease-centered prayer, (2) assurance-centered prayer, (3) God-centered prayer- reflecting the relationship between God and the patient, (4) others-centered prayer, and (5) lamentations- fear, doubt and anger in relation to God. Unsurprisingly disease centred prayer concerning an individual’s illness was found most frequently. The majority of patients prayed for an improvement in their disease, disease management or pain relief but it was actually quite rare to hope for or believe in cure. In other words they prayed for desirable but achievable outcomes. Especially among Muslims, patients preferred not to ask for cure for fear that their prayer would not be answered and to surrender to God’s will. Another common reason for praying was to receive the ability to find meaning or positive experiences in their illness.

Human suffering raises significant questions pertaining to the role of the Divine in the human world and might bring on a state of cognitive dissonance for believers based upon inconsistencies between the state of the world and religious beliefs ( Festinger, 1962). While requests for healing are commonplace, prayer requests may go unanswered. The sick person may not improve, deteriorate or even die. This raises significant issues as to why God does not answer prayer requests and is part of the wider question of theodicy: if God is omnipotent, omniscient and benevolent, why does suffering exist in the world? Unanswered prayer presents difficulties if believers maintain that God is omnipotent and all loving and can result in cognitive dissonance. But as the literature on cognitive dissonance demonstrates, religious believers frequently maintain or even intensify their convictions post disconfirmation (see Dein and Pargament, 2012; Tavris & Aronson, 2007). Such religious convictions are vigorously defended. Montell (2001) argues how religious belief is always held in tension with real world events:

‘Any and all active beliefs that transcendent forces give intrinsic meaning to life come into conflict with an unavoidable awareness of the harsh realities of natural history and current events, rampant human violence and cruelty, as well as natural disaster. Faced with that, one might ask how so many people educated in cause-and-effect reality, whose experience confirms that reality, maintain a belief in the Biblical God of creation, a just God who operates beyond the laws of causality? How can twenty-first century people, with empirical knowledge of recent world events, adhere to a belief in a prayer-answering personal saviour without incurring some discomfort? Whether or not such a God functions in some remote part of the universe, the empirical evidence increasingly indicates that no such God functions on earth. How can this seemingly impossible-to avoid logical inconsistency not lead to an impossible-to-maintain psychological inconsistency? Yet, somehow, belief in such a deity is maintained in alert and informed human minds. We have the ability to form and maintain beliefs that ameliorate difficult-to-accept, sensory-based experience and perception.’

Within the philosophical literature there is widespread discussion of these issues. Additionally many popular books have addressed the issue of unanswered prayer (e.g. Greig, 2011). Rather less have been written from a psychological perspective (see Dein, 2022 for an overview).

The above discussion assumes that if someone is not healed despite prayer, this creates a theological problem that must be psychologically resolved. Whether this is the case depends on one’s theological assumptions. The theologian Gregory Boyd (1997) explains that early Christians expected evil, personified in a literal devil and demons, and were accustomed to fighting against it; sickness was not a theological problem but an occasion to battle against the kingdom of Satan just as Jesus did: “The thief comes only to steal and kill and destroy; I have come that they may have life, and have it to the full” (John 10:10), “God anointed Jesus of Nazareth with the Holy Spirit and power, and how he went around doing good and healing all who were under the power of the devil, because God was with him" (Acts 10:38). Christians were thus admonished: “Do not give the devil a foothold” (Ephesians 4:27), “For we do not wrestle against flesh and blood, but against the rulers, against the authorities, against the cosmic powers over this present darkness, against the spiritual forces of evil in the heavenly places” (Ephesians 6:12). Likewise, even in the Old Testament, an angel said: “Do not be afraid, Daniel. Since the first day that you set your mind to gain understanding and to humble yourself before your God, your words were heard, and I have come in response to them. But the prince of the Persian kingdom resisted me twenty-one days. Then Michael, one of the chief princes, came to help me, because I was detained there with the king of Persia” (Daniel 10:12-13). Humans participated in a cosmic battle between God and his angels against Satan and his demons. This was fully expected, not a theological problem that needed to be solved through hermeneutical gymnastics to resolve cognitive dissonance.

Modern Christians, by contrast, do not expect evil, find it perplexing, resign themselves to it as God’s will, and try to understand it and resolve the psychological tensions their worldview creates, rather than fighting against evil. Boyd explains the difference between a “blueprint” and a “warfare” worldview. In the former, God is ultimately responsible for everything, including evil; unanswered prayer is therefore a problem because it raises questions about God’s goodness. People respond by looking for God’s purpose in sickness or disability. The warfare model instead explains evil as ultimately resulting from abuse of freedom by human or demonic agents. There is a cosmic war in which human and angelic agents have aligned themselves with either God or Satan. The battle is not eternal; it had a beginning point, the “fall,” and it will end since God’s victory was assured in Jesus’s triumph on the cross. The warfare worldview encourages hopeful action rather than learned helplessness.

The psychological theory of “learned helplessness” ( Seligman, 1972) can help to explain why many Christians do not pray for miraculous healing. Experimental psychology shows that when humans, as well as animals, experience an uncontrollable traumatic event, when later faced with controllable trauma they endure it passively even though they have the power to take effective action, and they show more stress in the face of trauma. Helplessness produces hopelessness. If subjects instead experience controllable trauma before uncontrollable trauma, they are more likely to continue to take action regardless of whether that action proves to be effective in a given situation, and they show less stress regardless of whether their actions are effective. When applied to prayer, it matters whether individuals have experiences, or hear about experiences, of healing through prayer before they find themselves in need of a miracle. Past disappointments or listening to sermons that teach an expectation of disappointment can result in learned helplessness and passivity in the face of disease or disability. Even a small experience of healing through prayer, or hearing other people’s testimonies of healing, produces hope and encourages persistent prayer even when prayers do not immediately result in healing.

## The 21st century Evangelicals research programme

The 21st century Evangelicals research programme is a quarterly online survey asks about various topics relevant to Christians ( Evangelical Alliance, 2023). Included in this survey are various questions related to health and more specifically prayer and God’s role in healing. The health survey conducted in 2015 included 1700 Evangelical Christian individuals. They derive from a wide variety of Protestant church denominations, Anglican, independent charismatic networks, Baptist, Free Church, Independent Evangelical and Pentecostal streams. The survey included multiple choice questions analysed through statistical means and open ended free text questions. Fifty one percent of the sample were from the older generation and 56.4% were male.

This paper deploys published data from Smith (2018) alongside the author’s analysis of Twenty First Century research programme. The data indicated that these Evangelical Christians asserted that God does intervene to heal miraculously (28% often; 70 % sometimes). There was widespread rejection of the idea that God always heals.

Participants mentioned several Biblical texts which informed their understandings of prayer and healing:

Psalm 103.2-3: Praise the LORD, my soul, and forget not all his benefits— who forgives all your sins and heals all your diseases,

Isaiah 53.5 By his stripes/wounds you have been healed. Quoted in 1 Peter 2.24,

James 5.14-15 Is anyone among you sick? Let them call the elders of the church to pray over them and anoint them with oil in the name of the Lord. And the prayer offered in faith will make the sick person well; the Lord will raise them up.

Of significant interest 86% deployed prayers asking that God will guide medical professionals and ensure their treatments were effective. The majority of respondents asserted that, while God can and does heal miraculously, this is frequently through means of medical interventions i.e. through naturalistic means: ‘I believe God heals through the work of doctors and medical treatment, difficult to separate divine healing from healing through medication and health services etc. through which God may work.’

Eighty three percent prayed for comfort in suffering. Significantly fewer (64%) prayed that God would heal. In other words prayers were less likely to pray for cure of their illness and more likely to pray for potentially achievable results. Other forms of healing were deployed including laying on of hands for healing (55%) and anointing with oil (26%).

Forty nine percent had received divine healing for an illness at some time in their lives. Examples included allergies, pain from fractured ribs, ME improved vision and no longer needing glasses, a burnt hand, self -harm and infertility. In a few instances the healings were extreme:’ My sisters baby girl was born blind but after prayer could see’. Those who had been healed emphasised that they had never suffered from the condition again:’Fifty years ago I was healed of gallstones and have had no recurrence of the illness since’. Often they mentioned that conventional medical treatments had failed before divine healing: ‘I was healed of a sports injury at New Wine. I could not race walk for nearly two years and physio did not help’. In other instances their testimonies focused upon how God had involved and guided medical staff.

## Unanswered prayer and cognitive dissonance

Around forty-nine percent of respondents mentioned at least on instance in which divine healing had failed and the sick person required further medical treatment or even died. Two examples: ‘My husband was not healed and needed his bowel removed’, ‘yes my sick grandfather dies just like God does not exist you know’. A wide range of views were expressed about unanswered prayer. These were rationalisations to avoid cognitive dissonance:

### The impossibility of understanding unanswered prayer

‘I don’t know why God heals some people and not others’

‘Its difficult to properly account for why healing clearly occurs on some occasions and not on others’

‘I can’t understand why we don’t see more miracles than we do’

‘One of the earliest lessons I learned is that people are healed differently’

‘Not everybody has been healed but there almost always reasons for this’

### God chooses to heal or not heal

‘Because healing would have been a blessing but God chose not to heal’

‘I believe God is sovereign and can heal but it is not always his main priority’

‘God is sovereign and we should not, and cannot, try to second guess or dictate to him’

‘God is sovereign –and his will is perfect. This means we should never be “disappointed”

‘Although we need to be faithful in praying and need to realise that the results are in God’s hands’

‘Not everyone we pray for gets healed but I encourage the person to continue praying since God is sovereign in all circumstances’

‘We pray in faith and hope but sometimes God chooses not to answer our prayers’

‘I praise God when he heals someone by ordinary or extraordinary means and I praise God when he chooses to not heal in this world and instead gives grace to live with problems or grace to die well and go to be with him forever.’

‘I believe that God sometimes heals today. I don’t believe that he always does so, even for people who really love him. I think the Church needs to find a middle way of providing prayer, care and support for those who are unwell, and a blame-free environment if they are not supernaturally healed.’

### God always does the best for us.

‘We ask God and he does what he knows is best’

### Having faith and discernment is important for healing

**‘**Recently my church was praying for a member who subsequently died. Looking back at this experience I sense that we were not in full agreement about the objective. For example, some were praying for successful medical treatment, some for the Lord’s “will” to be done. It was all a bit haphazard.’

‘At one time I used to pray and lay hands on the sick and wondered if God had given me a gift in this area. At that time I was praying for a friend’s mother who had cancer, really believing God would heal her, even as she appeared to become sicker. After she died it was a long time before I prayed for anyone in this way again and when I did it was not with a great amount of faith’

### Healing always occurs but is not always what we expect

‘Depends on expectations-there is always healing but not what we expect’

‘I believe when I pray for someone the healing has already began whether it is immediate or longer term’

‘Some people really do not want God to heal but ask for prayer. Others have still had the problem but were blessed in another way or were able to help others in similar circumstances. Only God knows the heart’

### Miracles no longer occur today

‘I believe the bible teaches that healing was for the Apostolic age and ceased with the completion of the canon of scripture. I believe God CAN heal if he wants to, but that he chooses to do so very rarely.’

## Discussion

In the Twenty First Century Evangelical sample, while the vast majority endorsed the idea that God could heal miraculously if he so chooses, the most frequent requests involved asking God to guide medical professionals and to ensure that their treatments were effective or to provide comfort and alleviate the suffering of the sick, rather than ask for a cure. Participants asked for desirable but achievable outcomes. To this extent these findings reflect the literature cited above reflecting healing through naturalistic rather than miraculous means (e.g. Dein, Stygall & Martin, 2006; Cadge & Daglian, 2008; Taylor *et al.*, 1999). We would argue that prayers need to conserve their relationship with the sacred and avoid cognitive dissonance ensuing from the incompatibility of two views: God is omnipotent and all loving and he has not answered my prayers. Unanswered payer raises issues of God’s ability or willingness to heal. Respondents deployed a range of theodicies to account for the failure of diving healing. These included perceived lack of faith in God’s healing ability, the fact that healing always occurs although it might not be what we expect, healing is not always immediate and may be longer term, the difficulty discerning answered prayer, People are healed differently, God not deciding to heal, God providing the grace to deal with pain and suffering even though a person is not physically cured, scepticism about miraculous healing and the view that miracles no longer occur after the Apostolic Age.

The findings can be tied to secularisation and disenchantment, the scientifically dominated society leaving little room for the interpretation of events in miraculous terms. Max Weber (1918-19) in his theory of disenchantment discusses how ‘mysterious and incalculable’ powers disappear from the world, nature is managed we may ask whether its social significance. Philosopher Charles Taylor (2007) discusses the fading of god’s mystery and providence and the overemphasis on science in understanding life. Unexplained healings are no longer understood in religious terms. The relationship between god and humanity is diminished. However we may ask whether there are alternative explanations for these findings?

Dein and Pargament (2012) propose that psychological rather than physical outcomes may be requested in order to avoid cognitive dissonance, to maintain the belief that God is fully able to heal if he so chooses and most importantly to conserve a relationship with the sacred (see also Dein, Stygall and Martin 2006; Cadge and Daglian 2008; Taylor 1999; Barrett 2001) ^.^ However as Pargament (1997) suggests, among non-conservative Christians in the USA, individuals look for strength and support from the Divine, rather than cure or miracle. Dein and Pargament (2012) assert that psychological outcomes as compared to physical outcomes can be more easily accomodated in God’s interventions in the process of healing. Boudry and De Smedt (2011) argue that psychosocial causation like relief from anxiety, anger or depression, possessing courage or mental strength, better coping etc are typically less observable and more complex, but more modest, in comparison to many forms of physical and biological causation. As these authors note, prayers for the sick might focus upon coping rather than cure since coping is less likely to fail compared with curing physical illness. As they state:

‘In other words, believers who request supernatural interventions that are subtle and indistinguishable from the natural course of events will have a better chance of finding themselves in a situation in which they can attribute the events in question to God answering their prayers.’

It is important to note in regard to the foregoing discussion that although the Twenty First Century Evangelical Sample offers insights into common theodicies, there are a number of important limitations in what this data can reveal. It obscures important differences among “evangelicals” included in the survey. One important distinction even among US and UK Christian who pray for healing (l *et al*one reflecting all of global pentecostalism) is between those who express a “blueprint” as opposed to a “warfare” ( Boyd, 1997) model of the nature of God’s sovereignty. The former model is exemplified by Calvinism ( Warfield, 1918) whereas the latter is reflected in offshoots of the Vineyard movement’s theology that the Kingdom of Heaven is both “already and not yet” ( Wimber & Springer, 1987; Clark, 1998) and that Christians are living in “in between times” and must consequently engage in a battle between kingdoms even though the ultimate victory was already won by Christ on the cross. If healing does not depend solely on the question of whether God “chooses” to answer prayer, then prayer involves not only petitions to God but also commands—to sicknesses and to any evil spirits or demons that might be causing diseases or other afflictions ( MacNutt, 1995), and “faith” may be understood more in terms of perseverance ( Brown, 2012) than an abstract and generally unfalsifiable cognitive capacity. Thus, it is important to note reasons why Christians who believe in healing might not pray for it while also recognizing that many Christians do pray for miraculous healing and believe that their prayers are answered. Many of these Christians acknowledge not understanding in every instance why some but not all are healed, but they are less likely to learn helplessness and thus resign themselves to sickness without a fight or give up as quickly when healing does not immediately result. They are also more likely to embrace the risk of disappointment and potential for emotional pain required in persevering prayer for miracles. ^1^

## Data Availability

The underlying data for this research is sourced from *Twenty-First Century Evangelicals, 2010-2016: Special Licence Access.* [data collection]. 4th Edition. UK Data Service. SN: 7786, DOI: http://doi.org/10.5255/UKDA-SN-7786-4.

## Footnotes

^1^
It is important to note that scholars of religion are slowly moving away from the notion of belief and doctrine towards embodiment and embodied experiences. This approach argues that representations of the Divine are grounded in an integrated and dynamic sensorimotor complex ( Soliman, Johnson & Song, 2015). This includes but is more than the brain. Religion is more than a set of beliefs, it is fundamentally a corporeal practice. Posture, gesture and movement, as part of worship, are closely intertwined with religious experience. The modification of bodily states can impact internal mental states. There has been a recent turn to ‘lived everyday religion’ ( McGuire, 2008; Ammerman, 2014; Jupp & Flanagan, 2007). Belief has been replaced by ‘spirituality’, ritual and embodiment and health is more closely tied to bodily experience.
