# Supplementary material for: Prayers for Sickness: What do people pray for and how do they deal with unanswered prayer?
Source: F1000Res. 2024 Sep 2;13:156. Originally published 2024 Mar 1. [Version 2] doi: 10.12688/f1000research.145194.2 (PMC11456154; doi:10.12688/f1000research.145194.2)
Supplement: Peer review history

# Reviewer report: Reviewer response for version 2

**DOI:** 10.5256/f1000research.170617.r357444

**Published:** 2025-01-25

**Authors:** Ahmet Tanhan

**Affiliations:** 1 The University of North Carolina, Greensboro, NC, USA

**Title of the work: Prayers for Sickness: What do people pray for and how do they deal with unanswered prayer?**

 Dear researcher(s), you are addressing an important and meaningful gap. Your paper is well-written and it has some important results, and if you edit your paper you can become much more effective. Here some humble suggestions to improve the paper, I would do the following to strengthen the paper. I have enjoyed reading the paper and am looking forward to seeing the paper indexed. You could increase the effect of your paper with some more recent studies suggested below or any other studies and not using the suggested ones. I have enjoyed reading the paper and please see my attached feedback.

 I humbly invite you to respond to my comments and revise your  manuscript. When you revise your manuscript please highlight the changes you make in the manuscript     by using the track changes mode in Word or by using coloured text; I would prefer coloured text. In this way I can see the changes much quicker.

**Main points: **

1. **Title**: good
2. **Abstract and keywords clear and comprehensive**: **good**
3. **Overall language**:

- The language is quite clear and well-written. You could use an active language for your future papers throughout the paper since an active language seems to be more effective. And more and more researchers go with an active language. However, you do not have to change for this paper- just a suggestion for your future work and I know some journals asking for a passive language.
- For an active language see suggested papers

 

1. **Length of paragraph **: good and
  1. you can check the paper and make sure every paragraph is not more than 5 sentences. The best is to stick with 3 to 5 sentences.
  2. And you can add some subtitles to be more organized and short sections. Please see the suggested papers above. I would suggest you to look at the suggested studies and use more subtitles to make the paper more organized.

 

1. **Introduction**: good and
  1. **You can use subtitles to make it more organized, you can check suggested studies**
2. **Thoroughness of the literature review:** can support with some recent studies yet do not have to. This will increase the effect of the paper and the journal.
3. **Clarity of the description of the Theoretical Framework (TF):** You do not have to construct a subsection called theoretical framework because you have enough explanation about which theories/perspectives have shaped your research. However, if you want to make your paper more effective I would construct a subsection called theoretical framework and briefly explains see suggested papers
4. **Research design: good**
5. **Clearly providing research questions and/or purpose: good and can have a clear subtitle to make it more visible**
6. **Method: Choice of research method:** very appropriate
7. **Appropriateness of procedures chosen for data collection and analysis:** good
8. **Results: Relevance of  data obtained in view of  the  purpose of the research:** good and it seems you have provided all the results under method section. You can check suggested studies to see how to shape your method and results.
9. **Discussion of the results and their significance:** good
10. **Soundness of conclusions in relation to data presented:** good
11. **Limitation**:  need to provide them under a clear subsection.
12. **Implication:**  good and
  1. you can increase the effect of your paper by constructing a new section entitled “implication” for clear and brief suggestions in at least two or three of the following most important to you mental health, education, research, administrators, services, etc.: see suggested papers  for implications for specific sections
  2. Your study is a mixed method study and you can call for future researchers to use some qualitative and innovative method to examine the same topic: using Online photovoice (OPV), Online Interpretative Phenomenological Analysis (OIPA) and Community-Based Participatory Research (CBPR) approach and act with and for people dealing with this subject for more grounded research to capture the thoughts, feelings, images, and behaviors of people from their own unique experiences to set the ground for more effective services, See suggested studies.
  3. I would strongly suggest you to call future researchers to use Online Photovoice (OPV), OIPA, and CBPR to conduct research on the same or similar topics: e.g., Using Online Photovoice (OPV) to understand the same topic you have addressed in your paper: using Online Photovoice (OPV) and collaborating with community to explore what prayer means to Christians, what they experience when they feel their prayers are unanswered.
  4. The researchers can use OPV, as one of the most recent and effective innovative qualitative research methods. Below I have provided some of the first OPV authors (e.g., Tanhan & Strack, 2020) who operationalized OPV and used and explained it step by step. OPV gives opportunities to the participants to express their own experience with as little manipulation as possible if at all, compared to traditional quantitative methods. As researchers one of our responsibilities is to inform others about recent and effective methods, which will increase the effect of your paper and the journal. Future researchers can conduct only qualitative or mixed method to see if OPV. And educators/trainers etc. also can use OPV for experiential activities to increase group and organizational synergy. OPV and OIPA allows one to use one of the most simple and straight approach to analyze the data in one of the most comprehensive and meaningful way. Please see suggested papers if you wish to do so.

You can get this first OPV paper from GoogleScholar or ResearchGate or you can search for other OPV studies through googlescholar

refer 1

  

1. **APA format:** good and please make sure you follow APA 7
  1. **You can get more information on APA 7 for free from this website** **https://owl.purdue.edu/owl/research_and_citation/apa_style/apa_formatting_and_style_guide/general_format.html**

  2. For information on APA style (7th edition) for references, please see:
    1. https://apastyle.apa.org/style-grammar-guidelines/references
    2.
**http://www.tandf.co.uk/journals/authors/style/reference/tf_APA.pdf**

I have enjoyed reading your paper and learned a lot- thanks for your contribution to social sciences. You are addressing an important and meaningful gap. Your paper has some important results, and if you edit your paper based on all or some of the humble suggestions above, it can be much more effective. I am looking forward to seeing the paper indexed. You could increase the effect of your paper with some more recent studies suggested above.

Is the work clearly and accurately presented and does it cite the current literature?

Yes

If applicable, is the statistical analysis and its interpretation appropriate?

Yes

Are all the source data underlying the results available to ensure full reproducibility?

Yes

Is the study design appropriate and is the work technically sound?

Partly

Are the conclusions drawn adequately supported by the results?

Yes

Are sufficient details of methods and analysis provided to allow replication by others?

Yes

Reviewer Expertise:

counseling, psychopathology, spirituality

I confirm that I have read this submission and believe that I have an appropriate level of expertise to confirm that it is of an acceptable scientific standard, however I have significant reservations, as outlined above.

## References

[1] : Online photovoice to explore and advocate for Muslim biopsychosocial spiritual wellbeing and issues: Ecological systems theory and ally development. *Current Psychology* .2020;39(6) : 10.1007/s12144-020-00692-6 2010-2025 10.1007/s12144-020-00692-6

# Reviewer report: Reviewer response for version 2

**DOI:** 10.5256/f1000research.170617.r359589

**Published:** 2025-01-22

**Authors:** Rikas Saputra, Rizky Andana Pohan

**Affiliations:** 1 Universitas Islam Negeri Raden Fatah Palembang, Palembang, South Sumatra, Indonesia; 2 Islamic of Guidance and Counselling, IAIN Langsa (Ringgold ID: 524734), Langsa, Aceh, Indonesia

**Expert Review Report**

**Article Title:**

 Prayers for Sickness: What Do People Pray for and How Do They Deal with Unanswered Prayer?

**Authors:**

 Simon Dein, Candy Gunther Brown

**Article Summary:**

 This article examines the use of prayer in the context of illness, focusing on the content of prayers, expectations of healing, and how individuals deal with unanswered prayers. Secondary data were drawn from the Twenty-First Century Evangelical research program, a survey covering 1,700 Christians in the UK. Key findings include prayer preferences for medical comfort and guidance over immediate healing and theological and psychological rationalizations for unanswered prayers, such as cognitive dissonance theory and learned helplessness. This article emphasizes the importance of prayer in creating a relationship with God and avoiding disappointment.

**Evaluation Based on Review Questions**

 1. Is the work presented clearly and accurately, and does it cite current literature?

**Answer:** Partially.

**Explanation:**

 The article is logically organized and uses relevant literature, but some references are not current. Most of the primary literature, such as the systematic review by Roberts et al. (2009), is over 10 years old. In addition, the study’s cultural context was limited to the Evangelical Christian community in the UK, which may reduce the generalisability of the results.

**Suggestions for Improvement:**

1. Add recent references, particularly studies after 2015, to strengthen the relevance of the findings.
2. Expand the scope of the literature to include other religious communities or different geographical regions, thus enriching the analyses.

2. Was the research design appropriate, and was the work technically sound?

**Answer:** Partially.

**Explanation:**

 The article used survey data with thematic analyses, but there was no triangulation or validation of findings by a third party. Analyses were conducted by one individual, with no thematic reliability checks.

**Suggested Improvement:**

1. Use two or more researchers to review the qualitative data, increasing the reliability of the results.
2. Add a detailed explanation of how themes were coded and analyzed.
3. Include primary data to strengthen the validity of the findings.

3. Are the details of the methods and analyses provided sufficient to allow replication by others?

**Answer:** Partially.

**Explanation:**

 The article describes thematic analysis methods but does not include specific procedures such as tools or software used for data analysis.

**Suggested Improvement:**

1. Describe the thematic analysis steps, including the software (e.g., NVivo or ATLAS.ti) used.
2. Include tables or diagrams to demonstrate the analysis process and themes identified.

4. If applicable, is the statistical analysis and interpretation appropriate?

**Answer:** Not applicable.

**Explanation:**

 The article does not use statistical analyses, as the focus is on qualitative data. However, the interpretation of the descriptive data from the survey is entirely consistent with the findings.

 5. Is all source data underlying the results available to ensure reproducibility?

**Answer:** Partially.

**Explanation:**

 The survey data came from the UK Data Service, but the complete data was unavailable directly in the article. This limits the reader’s ability to replicate the results.

**Suggested Improvement:**

1. Attach examples of survey data or interview transcripts as appendices or additional links.
2. Explain the procedure for accessing the dataset, including any limitations on its use.

6. Are the conclusions drawn adequately supported by the results?

**Answer:** Partially.

**Explanation:**

 The survey data directly supports the conclusion that prayer is more likely for medical comfort and guidance than healing. However, the generalisability of the findings is limited as the sample comes from only one geographical area.

**Suggested Improvement:**

1. Expand the analysis to include comparisons with other communities, such as non-Christian religions or Christian denominations.
2. Discuss potential cultural bias and how it affects the results.

**Overall Recommendation**

 This article provides essential insights into the role of prayer in the context of disease but needs improvement in the following aspects:

1. Adding current literature to increase academic relevance.
2. Using triangulation or validation of findings by more than one analyst.
3. Providing details of thematic analysis procedures to increase transparency and replicability.
4. Expand geographic or community coverage to strengthen the generalisability of results.
5. Attach sample data or dataset access procedures to ensure reproducibility.

Is the work clearly and accurately presented and does it cite the current literature?

Partly

If applicable, is the statistical analysis and its interpretation appropriate?

Not applicable

Are all the source data underlying the results available to ensure full reproducibility?

Partly

Is the study design appropriate and is the work technically sound?

Partly

Are the conclusions drawn adequately supported by the results?

Partly

Are sufficient details of methods and analysis provided to allow replication by others?

Partly

Reviewer Expertise:

Counselling and mental health

We confirm that we have read this submission and believe that we have an appropriate level of expertise to confirm that it is of an acceptable scientific standard.

## References

[1] : Religion, spirituality, and health: the research and clinical implications. *ISRN Psychiatry* .2012;2012: 10.5402/2012/278730 278730 10.5402/2012/278730 23762764 PMC3671693

# Reviewer report: Reviewer response for version 2

**DOI:** 10.5256/f1000research.170617.r319844

**Published:** 2024-10-17

**Authors:** Dick Kruijthoff

**Affiliations:** 1 Vrije Universiteit Amsterdam, Amsterdam, North Holland, The Netherlands

The texts which were added by the authors are adequate.

 The additional information on the methodology used is relevant by now mentioning that the data were analysed through thematic analysis, with a limitation of the research as only one person did the analysis. This is essential information. In future research of the kind It would be advisable to have at least 2 persons doing the analysis. 

 I fully agree with the addtional text in the conclusion section, stressing the holistic character of these healings and the need to include other fields of knowledge apart from biomedicine. My only suggestion here is to name this section conclusion and suggestions for future research **(or something similar), since there is an emphasis on suggestions for future work in this area.

 Finally I wish the authors inspiration and enthusiasm for further research in this understudied field.

Is the work clearly and accurately presented and does it cite the current literature?

Yes

If applicable, is the statistical analysis and its interpretation appropriate?

Not applicable

Are all the source data underlying the results available to ensure full reproducibility?

Yes

Is the study design appropriate and is the work technically sound?

Yes

Are the conclusions drawn adequately supported by the results?

Partly

Are sufficient details of methods and analysis provided to allow replication by others?

Partly

Reviewer Expertise:

Study of Healing after Prayer at VU university Amsterdam (as MD)

I confirm that I have read this submission and believe that I have an appropriate level of expertise to confirm that it is of an acceptable scientific standard.

# Reviewer report: Reviewer response for version 2

**DOI:** 10.5256/f1000research.170617.r319845

**Published:** 2024-10-04

**Authors:** Elizabeth Taylor

**Affiliations:** 1 Loma Linda University, Loma Linda, California, USA

Thank you for some added information regarding the methods. This report should be helpful to those wanting to better understand the thinking of Christians who perceive unanswered prayers.

Is the work clearly and accurately presented and does it cite the current literature?

Partly

If applicable, is the statistical analysis and its interpretation appropriate?

Not applicable

Are all the source data underlying the results available to ensure full reproducibility?

No

Is the study design appropriate and is the work technically sound?

Partly

Are the conclusions drawn adequately supported by the results?

Yes

Are sufficient details of methods and analysis provided to allow replication by others?

No

Reviewer Expertise:

Spirituality/religiosity and health, nursing and spiritual care

I confirm that I have read this submission and believe that I have an appropriate level of expertise to confirm that it is of an acceptable scientific standard.

# Reviewer report: Reviewer response for version 1

**DOI:** 10.5256/f1000research.159105.r293332

**Published:** 2024-06-27

**Authors:** Elizabeth Taylor

**Affiliations:** 1 Loma Linda University, Loma Linda, California, USA

This research report shares secondary findings from a quarterly online survey of Evangelical Christians that inform about what their perspectives are regarding unanswered prayers for health (presumably physical and psychological). 

 * The strength of this reporting is in the very rich bookends: the review of literature and theory to introduce the findings, and the Discussion. These clearly show the authors are well-versed and think deeply on the topic--indeed, a relevant and highly interesting and helpful topic.

 * My only complaint, and it is a serious one, is that the Methods are not adequately described. Tell us more about how the survey was conducted and where. Were the empirical indicators validated and reliable? How was the qualitative data generated and analyzed? Did a theoretical framework guide that analysis? Were any measures instituted to insure trustworthiness of the qual data/findings?

 * Reflections: This article reminds me that the 21st century priest is the psychologist. Science is superseding theology, it seems. As former NIH Director and Templeton Prize winner Francis Collins would say, however, God's word (holy scripture, inspirations) and God's ways (creation, the natural world that science studies) are both valid. 

 * The blueprint vs warfare worldviews were helpful to cite. Carl Jung's thinking about God enveloping evil/darkness is pertinent to the blueprint thinking.

Is the work clearly and accurately presented and does it cite the current literature?

Partly

If applicable, is the statistical analysis and its interpretation appropriate?

Not applicable

Are all the source data underlying the results available to ensure full reproducibility?

No

Is the study design appropriate and is the work technically sound?

Partly

Are the conclusions drawn adequately supported by the results?

Yes

Are sufficient details of methods and analysis provided to allow replication by others?

No

Reviewer Expertise:

Spirituality/religiosity and health, nursing and spiritual care

I confirm that I have read this submission and believe that I have an appropriate level of expertise to confirm that it is of an acceptable scientific standard, however I have significant reservations, as outlined above.

# Response

**Published:** 2024-08-13

**Authors:** Simon Dein

**Affiliations:** Psychiatry, Queen Mary University of London, London, England, UK

reviewer 2 

 She has commented upon lack of detail in methodology

 We have rewritten this 

 The 21st century Evangelicals research programme is a quarterly online survey asks about various topics relevant to Christians ( Evangelical Alliance, 2023). Included in this survey are various questions related to health and more specifically prayer and God’s role in healing. The health survey conducted in 2015 included 1700 Evangelical Christian individuals. They derive from a wide variety of Protestant church denominations, Anglican, independent charismatic networks, Baptist, Free Church, Independent Evangelical and Pentecostal streams. The survey included multiple choice questions analysed through statistical means and open ended free text questions.

**The sample resided in the UK. The qualitative data were analysed through thematic analysis using spreadsheets to sort the data. Common themes were elicited.  The quotations that appear in the paper are chosen because they are in some way typical of common experiences and interesting in themselves.  The questions in the survey were validated by a panel of academic experts. A limitation of the qualitative data is that it was analysed by only one person and no attempt was made to look at the reliability of themes. The fill dataset can be found at SN 7787 - Twenty-First Century Evangelicals, 2010-2016**

**https://discover.ukdataservice.ac.uk/catalogue/?sn=7787&type=Data%20catalogue.**

**We have included a brief discussion of Jung as suggested**

**Modern Christians, by contrast, do not expect evil, find it perplexing, resign themselves to it as God’s will, and try to understand it and resolve the psychological tensions their worldview creates, rather than fighting against evil. One of the best-known modern psychological efforts was suggested by Carl Jung (1954). He proposed that God incarnated as a purely good redeemer only after self-realization of having unjustifiably tormented Job under provocation by Satan. Boyd explains the difference between a “blueprint” and a “warfare” worldview, neither of which embraces Jung’s psychological analysis of God’s evil side**.

# Reviewer report: Reviewer response for version 1

**DOI:** 10.5256/f1000research.159105.r257992

**Published:** 2024-04-22

**Authors:** Dick Kruijthoff

**Affiliations:** 1 Vrije Universiteit Amsterdam, Amsterdam, North Holland, The Netherlands

The article addresses relevant questions relating to prayers for sickness and healing.

 It is a subject drawing a lot of attention among Christians and among the entire population as well. The authors thereby distinguish a 'blueprint' worldview from a 'warfare' worldview among Christians, which I consider to be a very adequate distinction as the two poles clearly reflect the different opinions. The differing views are highlighted by quotes from the 'Evangelical research programme', which increases the imagination, Both because of its relevance and the approach used I consider this article of significant interest.

 I have listed a few remarks and suggestions below:

 - The **Methods** paragraph in the abstract partly seems to reflect some results in stead of methods (as from 'the data suggest.....'). The authors may rearrange these elements of the text.

 - In the section **What do believers ask God for.... **the authors noticed that, based on the psychological theory of learned helplessness, a 'blueprint' approach towards prayer may eventually produce hopelessness. While the 'warfare' worldview encourages hopeful action. I agree with the former, but in the case of the 'warfare' approach one may also think of frustration and disappointment because of unanswered prayers, in due course leading to bitterness. Did the authors find articles on such adverse reactions when reviewing the literature? If so, it may be helpful to mention some of it.

 - A small separate **Methods **section in the article itself is recommended, In the abstract this is already mentioned, where the authors indicate that the methodology is based on a critical literature review and on secondary data from the Evangelical research programme. Which literature is included (and which is not)? Was there  a search strategy? As for the quotes deployed from the research programme one wonders if all quotes relating to the avoidance of cognitive dissonance were included. Or was it a part of the quotes? If so, was a strategy used when selecting the quotes?

 - In the **Discussion **the authors return to the 'blueprint' and the 'warfare' worldview, the former being exemplified bij Calvinism and the latter by charismatic movements such as Vineyard. In this respect it may be also interesting to include some of the lines of thougth in the Reformed (calvinist)- Pentecostal dialogue. Van der Kooi, a Reformed theologian, refers to the dialogue in his book 'This incredibly benevolent force' (Kooi van der, 2018). He considers the Pentecostal and charismatic movements to be a challenge for Reformed theology, with their (renewed) emphasis on gifts of the Spirit such as healing, prophecies, dreams and visions, empowerment. In his view there is not only a line from dogmatics to practice, but also from practice to dogmatics: 'We need to take seriously the experiences of Christians and churches in our own context, as well as of those on other continents and in culturally very different contexts' (page 19). According to this reciprocal relationship between theology and reality (or practice) 'the Reformed-Pentecostal dialogue drew a wider conclusion, in which other means of revelation were mentioned, aside from the Bible and sermons alone' (page 132). Coming back to 'blueprint' and 'warfare' views dilemmas between them may be resolved, at least to some extent, when studying and investigating practices. Brown pleads for the use of empirical methods to test for the effects of prayer on health (Brown, 2015). What actually happens in reality and what does not happen? Some of it is quoted by the authors (Romez, 2019, 2021; Brown, 2012; Roberts et al., 2009). In other articles (Austad et al., 2020; Helming, 2011; Francois et al., 2014; Kruijthoff et al., 2022) there appears to be a frequent association between healings related to prayer(s) on the one hand and 'Spirit' manifestations on the other hand such as strong sensory manifestations, extrasensory phenomena (prophecies, visions), transformation and empowerment. These 'holistic' healings, often occurring instantaneously, appear to be quite different from what we usually see in medical practice. In a fair number of instances they are being reported as having occurred unexpectedly, without much 'warfare'. When facing controversies between differing views among Christians empirical studies may indeed help us to move forward. Experiential data can thereby increase our understanding, apart from medical data. It is a challenge if  the authors consider to include some of these elements in the discussion.

Is the work clearly and accurately presented and does it cite the current literature?

Yes

If applicable, is the statistical analysis and its interpretation appropriate?

Not applicable

Are all the source data underlying the results available to ensure full reproducibility?

Yes

Is the study design appropriate and is the work technically sound?

Yes

Are the conclusions drawn adequately supported by the results?

Partly

Are sufficient details of methods and analysis provided to allow replication by others?

Partly

Reviewer Expertise:

Study of Healing after Prayer at VU university Amsterdam (as MD)

I confirm that I have read this submission and believe that I have an appropriate level of expertise to confirm that it is of an acceptable scientific standard.

## References

[1] : Reinscribing the Lived Body: A Qualitative Study of Extraordinary Religious Healing Experiences in Norwegian Contexts. *Religions* .2020;11(11) : 10.3390/rel11110563 10.3390/rel11110563

[2] : The Lourdes medical cures revisited. *J Hist Med Allied Sci* .2014;69(1) : 10.1093/jhmas/jrs041 135-62 10.1093/jhmas/jrs041 22843835 PMC3854941

[3] : Healing through prayer: a qualitative study. *Holist Nurs Pract* .2011;25(1) : 10.1097/HNP.0b013e3181fe2697 33-44 10.1097/HNP.0b013e3181fe2697 21150503

[4] : Prayer and Healing: A Study of 83 Healing Reports in the Netherlands. *Religions* .2022;13(11) : 10.3390/rel13111056 10.3390/rel13111056

[5] : Cornelisvan derKooi, This Incredibly Benevolent Force: The Holy Spirit in Reformed Theology and Spirituality. Grand Rapids: Eerdmans, 2018, xviii + 158pp., $38.00. *International Journal of Systematic Theology* .2019;21(1) : 10.1111/ijst.12322 97-100 10.1111/ijst.12322

[6] : Pentecostal Healing Prayer in an Age of Evidence-Based Medicine. *Transformation: An International Journal of Holistic Mission Studies* .2015;32(1) : 10.1177/0265378814537750 1-16 10.1177/0265378814537750

# Response

**Published:** 2024-08-13

**Authors:** Simon Dein

**Affiliations:** Psychiatry, Queen Mary University of London, London, England, UK

We have cited the references you suggest 

 There is a methods section at the start of the paper

 The paper is based upon on a critical literature review and deploys secondary data from the Twenty First Century Evangelical research program which includes a quantitative and qualitative component. The qualitative component involved open ended questions which were analyzed through thematic analysis.  

 We have added a brief discussion of holistic healing and 'spirit manifestations'  in our conclusion 

**What might future work in this area involve? To date much of the research on prayer and sickness has focused upon the medical rather than experiential aspects ie what are the medical outcomes. But often the healing is more holistic involving physical, mental and spiritual changes. And how are these experiences related together?  How do the biological and existential blend? And to what extent can spiritual experiences have a placebo effect?**

**These 'holistic' healings, often occurring instantaneously, differ from what we usually see in medical practice and reported as having occurred unexpectedly, without much 'warfare'. These include strong sensory manifestations, sense of God’s presence; extrasensory phenomena (prophecies, visions), a sense of being overwhelmed,  transformation and empowerment ( Romez, 2019, 2021; Brown, 2012; Roberts et al., 2009; Austad et al., 2020; Helming, 2011; Francois et al., 2014; Kruijthoff et al., 2022; Bendien et al 2023).  Some of these studies derive from a Northern European context. Religious and spiritual encounters like visions and voices may have a transformative function which some authors describe as healing (Braud 2012; Nygaard et al 2017). They can be life changing.  Healings related to prayer(s) on the one hand and 'Spirit' manifestations appear to be strongly related.**

**We would assert that a focus upon the ‘lived body’ and experiential data  -** **reports of their sensations, feelings, thoughts, and imagery before, during, or after a healing event -** **can thereby increase our understanding of religious healing through prayer, apart from medical data.  To conclude, the efficacy of prayer cannot be judged solely by biomedical criteria. Prayer might impact emotional and spiritual aspects, and this influences how those praying conceptualize its efficacy. We would agree with the authors cited above who argue for a horizontal epistemology in which** **all relevant perspectives are valuable, including both experiential and objective data. Future studies of prayer and health should be interdisciplinary-medical, psychological and theological.**
